# Effects of Silver Diamine Nitrate and Silver Diamine Fluoride on Dentin Remineralization and Cytotoxicity to Dental Pulp Cells: An In Vitro Study

**DOI:** 10.3390/jfb13010016

**Published:** 2022-02-01

**Authors:** Surapong Srisomboon, Matana Kettratad, Andrew Stray, Phakkhananan Pakawanit, Catleya Rojviriya, Somying Patntirapong, Piyaphong Panpisut

**Affiliations:** 1 Department of Oral Health Care, Lamlukka Hospital, Pathum Thani 12150, Thailand; surapong.sri@dome.tu.ac.th; 2 Faculty of Dentistry, Thammasat University, Pathum Thani 12120, Thailand; pmatana@staff.tu.ac.th (M.K.); p_somying@hotmail.com (S.P.); 3 DentaLife, Ringwood, VIC 3134, Australia; astray@dentalife.com.au; 4 Synchrotron Light Research Institute (Public Organization), Nakhon Ratchasima 30000, Thailand; phakkhananan@slri.or.th (P.P.); catleya@slri.or.th (C.R.); 5 Thammasat University Research Unit in Dental and Bone Substitute Biomaterials, Thammasat University, Pathum Thani 12120, Thailand

**Keywords:** silver diamine nitrate, silver diamine fluoride, dental caries, tooth demineralization, tooth remineralization, cytotoxicity test, dental pulp, synchrotron, X-ray microtomography

## Abstract

Silver diamine nitrate (SDN) is expected to help control caries similar to silver diamine fluoride (SDF). The aim of this study was to determine the mineral precipitation in demineralized dentin and the cytotoxicity of SDN and SDF to dental pulp cells. Demineralized dentin specimens were prepared, and SDF, SDN, or water (control) was applied. The specimens were then remineralized in simulated body fluid for 2 weeks. The mineral precipitation in the specimens was examined using FTIR-ATR, SEM-EDX, and synchrotron radiation X-ray tomographic microscopy (SRXTM). Additionally, the cytotoxicity of SDF and SDN to human dental pulp stem cells was analyzed using an MTT assay. The increase in FTIR spectra attributable to apatite formation in demineralized dentin in the SDF group was significantly higher compared to the SDN and control groups (*p* < 0.05). Dentinal tubule occlusion by the precipitation of silver salts was detected in both SDF and SDN groups. The mineral density as shown in SRXTM images and cytotoxicity of both SDN and SDF groups were comparable (*p* > 0.05). In conclusion, SDF demonstrated superior in vitro apatite formation compared to SDN. However, the degree of mineral precipitation and cytotoxic effects of both were similar.

## 1. Introduction

Untreated dental caries represent the most common preventable chronic disease affecting people of all ages worldwide [1]. A study showed that at least 1 in 5 adults in the U.S. population have untreated caries [2]. Current cost-effective cavity management consists of delaying irreversible surgical treatment and promoting remineralization to arrest the progression of lesions [3]. Low-invasive methods are also suitable for patients with special needs or with limited cooperation. The most common non-invasive materials for controlling dental caries are professionally applied fluoride materials such as silver diamine fluoride (SDF) [4] and NaF varnish [5]. The use of SDF is a cost-effective method of arresting dental caries [6,7]. It was demonstrated that biannual application of SDF led to a higher level of prevention of caries progression than NaF varnish [8].

There are four main anti-caries effects from SDF. The first is direct antibacterial action from the silver ion of SDF [9]. The second effect is the precipitation of silver phosphate or silver chloride [10], which can potentially enhance lesion hardness and act as a protective layer against dental biofilm. The third action is the formation of low-soluble and acid-resistant fluorohydroxyapatite, which can increase resistance to caries for the tooth surface. The fourth action is the ability to preserve collagen in dentin, which is essential for mineral precipitation. Silver ion was shown to reduce the degradation of collagen, which acts as a template for mineral precipitation [11]. The SDF solution can be rapidly adsorbed into dentin. The concern was that the high level of reactive ions in SDF could induce cytotoxic effects on the dentin–pulp complex. It was demonstrated that reactive ions such as Ag ion could diffuse into dentin up to 5–40 μm [12]. It was reported that the toxic effects of Ag and F ions are depleted glutathione and increased oxidative stress or lipid peroxidation [13]. This leads to reduced antioxidant properties, resulting in cell death and inflammation. Additionally, it was reported that the cytotoxic action of hydroxyapatite disc treated with SDF persisted even after 77 days of water rinsing [14]. A study also proposed applying glutathione with SDF to promote antioxidant functions and decrease the toxic effects from SDF on dental pulp cells [13].

An alternative silver solution for controlling caries is silver nitrate (AgNO_3_). An in vitro study showed that the application of AgNO_3_ or AgF increased the mineral density of demineralized enamel and dentin [15]. The increased mineralization was believed to be mainly due to the deposition of silver ions. Additionally, many studies have demonstrated that the use AgNO_3_ in combination with NaF varnish had comparable effectiveness in caries prevention compared to SDF [16,17,18,19]. This method may be more feasible, with a lower cost, compared to SDF, since AgNO_3_ solution and NaF varnish are already available in many countries [20]. However, the concern with AgNO_3_ is the delay in mineral induction time, which may affect remineralization in dentin [21]. SDF contains diamine groups, which may enable the formation of NH_4_OH, which could potentially help promote suitable pH and conditions for mineral formation and enhance antibacterial action [22,23]. The addition of diamine groups to stabilize silver ions in AgNO_3_ nitrate solution, forming silver diamine nitrate (SDN), is expected to help enhance the mineral precipitation of the solution.

Although SDF is considered a cost-effective intervention for controlling caries, the cost of the materials can vary across regions. Additionally, SDF may still not be available in some countries [24]. The cost of SDF in the U.S. was approximately USD 30–52 per application [6]. From the manufacturer’s point of view, the cost of an alternative silver solution such as silver diamine nitrate (SDN) is expected to be lower than that of SDF due to the lack of fluoride components. This would help reduce the economic burden for cavity prevention programs [25]. Currently, in vitro assessment of the remineralizing effects of SDN is limited. The aim of the current study was, therefore, to compare the mineral precipitation in demineralized dentin and the cytotoxicity of pulp cells between silver diamine nitrate (SDN) and silver diamine fluoride (SDF). It was expected that SDN would encourage mineral precipitation similar to SDF, and that the cytotoxic effects of SDN on dental pulp cells would be comparable with those of SDF. The null hypothesis was that the in vitro mineral precipitation and relative cell viability between SDF and SDN would not be significantly different.

## 2. Materials and Methods

### 2.1. Specimen Preparation for Remineralizing Studies

Extracted human third permanent molars of comparable size and with no visible cavitated carious lesions were collected from the Department of Oral Health Care, Thammasat University Hospital, Pathum Thani, Thailand. The use of human teeth was approved by the Ethics Review Sub-Committee for Research Involving Human Research Subjects at Thammasat University (approval number: 150/2562). The teeth were stored for less than 30 days in 0.1% thymol solution (M-Dent, Faculty of Dentistry, Mahidol University, Bangkok, Thailand) at 23 °C prior to the experiment.

Specimen preparation was performed according to the protocol used in the previous study [26]. Briefly, the teeth were embedded in self-curing acrylic resin (*n* = 7). The crown of each tooth was sectioned horizontally and perpendicular to dentinal tubules. The diamond blade of the cutting machine (Accutom 50, Struers, Cleveland, OH, USA) was positioned at ~2 mm below the occlusal surface. The obtained dentin slices (2.0 ± 0.1 mm thick) were then polished with microfine 4000-grit abrasive paper in a polishing machine (Tegramin, Struers, Cleveland, OH, USA). Then, the specimens were cleaned in an ultrasonic bath for 5 min. Each dentin slice was cut into 3 pieces using a greater taper medium-fine diamond bur to produce a total of 21 dentin specimens.

The specimens were demineralized in 17% ethylenediamine tetraacetic acid (EDTA; Faculty of Dentistry, Chulalongkorn University, Bangkok, Thailand) for 72 h to produce completely demineralized layers (depth of ~500 μm) [27,28]. Then, 25 μL of silver diamine nitrate (SDN) solution (48% SDN; Dentalife, Victoria, Australia), SDF (38% SDF, Topamine^TM^; Dentalife, Victoria, Australia), or deionized water (control group) was applied to specimens from each tooth (*n* = 7/group) for 30 s. The specimens were then cleaned with water from a triple syringe for 10 s, and immersed in simulated body fluid (SBF; BS ISO 23317:2014) (Table 1) [29]. SBF contains the same phosphate concentration as blood plasma or body fluid (pH = 7.40) (Table 2) [30,31]. SBF was expected to mimic the environment where the solution was adsorbed into the dentin and exposed to dentinal fluid. The specimens were incubated at 37 °C for up to 2 weeks without replacing the solution.

### 2.2. Assessment of Apatite Precipitation Using FTIR and SEM-EDX

Apatite formation on the demineralized dentin was examined using a Fourier transform infrared spectrometer equipped with attenuated total reflection (FTIR-ATR; Nicolet iS5, Thermo Fisher Scientific, Waltham, MA, USA) (*n* = 7) [26,32,33,34,35]. FTIR spectra in the region of 700–4000 cm^−1^ (resolution of 8 cm^−1^ with 12 repetitions) were recorded from the bottom surface of the specimen. The FTIR spectra of specimens were recorded after demineralization, then after remineralizing in SBF for 1 day, 1 week, and 2 weeks.

The ratio of FTIR area attributed to hydroxyapatite (1024 cm^−1^, PO_4_^3−^ stretch) [36] over the peak representing type I collagen in dentin (1636 cm^–1^, C=O stretch of amide I) [37] was obtained using OMNIC Series software (Thermo Fisher Scientific, Waltham, MA, USA). The mineral/matrix ratio (Abs_1024_/Abs_1636_) was then calculated. An increase in the Abs_1024_/Abs_1636_ ratio was expected to relate to an increase in mineral precipitation (remineralization) in demineralized dentin [26].

A representative specimen from each group was then selected to assess the mineral precipitation on the surface. The specimens were coated with gold in a sputter-coating machine (Q150R ES, Quorum Technologies, East Sussex, UK) using a 23 mA current for 45 s. A dispersive X-ray spectrometer (EDX, X-Max 20, Oxford Instruments, Abingdon, UK) was employed to analyze the elemental composition of precipitation on the specimens. The EDX spectrum was obtained from the precipitate using magnification of 20,000× and beam voltage of 5 kV. Data were then analyzed using INCA software version 5.05 (ETS, Stuttgart, Germany).

### 2.3. Assessment of Mineral Precipitation Using Synchrotron-Based X-ray Tomography (SRXTM)

Representative specimens (*n* = 3) at 2 weeks were selected and blotted dry (*n* = 3). The mineral density in the demineralized area was examined by a synchrotron X-ray source, Beamline 1.2 W X-ray imaging and tomographic microscopy (XTM), according to the method used in a previous study [26]. The synchrotron X-ray radiation originated from a 2.2-Tesla multipole wiggler at the Siam Photon Source operated at 1.2 GV. By using a polychromatic X-ray beam with a distance from source to sample of 32 m, the experiments were executed at a mean energy of 14 kV. Representative specimens were mounted on the stage. Then, X-ray radiographs were collected from 0° to 180° with an angular increment of 0.2°. The collected X-ray radiographs were then analyzed using Octopus Reconstruction software (TESCAN, Gent, Belgium) [38] to produce reconstruction images. After obtaining the reconstruction images, the degree of mineral precipitation was calculated by using Octopus Analysis software. In this case, 200 reconstruction images (or 288 μm) were chosen and averaged by 3 random areas (~10 × 10 μm). The reconstruction images were computed by using Drishti software [39] to produce the 3D tomographic reconstruction.

### 2.4. Cytotoxicity Test

Human dental pulp stem cells (hDPSCs) were obtained from Lonza (PT-5025, Group AG, Basel, Switzerland). Cells were maintained in Dental Pulp Stem Cell Basal Medium supplemented with Dental Pulp Stem Cell Growth Supplement, L-glutamine, ascorbic acid, and gentamycin/amphotericin-B (all from Lonza) at 37 °C enriched with 5% CO_2_. For the experiment, hDPSCs at passage 3 were switched to culture in Dulbecco’s Modified Eagle Medium (Sigma-Aldrich, St. Louis, MO, USA) with 10% fetal bovine serum and 1% penicillin/streptomycin and seeded in a 96-well plate with a cell density of 5,000 cells/well. The cells were then treated with 25 μL of SDF or SDN. Cells with no treatment were used as the control. The cells were cultured at 37 °C enriched with 5% CO_2_ for 3 days. Then, an MTT viability assay was performed. DPSCs were incubated with 0.2% 3-(4,5 dimethylthiazolyl)-2,5-di-phenyltetrazolium bromide (MTT) solution (Sigma-Aldrich, St. Louis, MO, USA) at 37 °C for 4 h. The reaction was paused using 200 μL of dimethylsulfoxide (Sigma-Aldrich, St. Louis, MO, USA) and 25 μL glycine buffer (Research Organics, Cleveland, OH, USA). The color of the end product was quantified using absorbance at 620 nm [40,41] under a spectrophotometer (Sunrise Absorbance Microplate Reader, Tecan Group Ltd., Männedorf, Switzerland). The results were expressed as relative optical density (OD) at 620 nm using the following equation:

(1) $$\mathrm{Relative}\;\mathrm{OD}=\frac{\mathrm{OD of test group}}{\mathrm{OD of control}}\times 100$$

### 2.5. Statistical Analysis

Data were analyzed using Prism 9 for macOS (GraphPad Software, San Diego, CA, USA). The normality of data was initially examined using the Shapiro–Wilk test. Changes in Abs_1024_/Abs_1636_ for the same group upon immersion time were compared using one-way repeated ANOVA followed by Tukey’s multiple comparisons. Differences in Abs_1024_/Abs_1636_ and mineral density were determined using one-way ANOVA followed by Tukey’s multiple comparisons. Additionally, the unpaired t-test was used to compare relative OD values between SDF and SDN groups. All *p*-values below 0.05 are considered statistically significant. A post hoc power analysis was performed using G*Power version 3.1.9.6 (University of Dusseldorf, Dusseldorf, Germany). The effect size [42] of each experiment was calculated from the results obtained from the previous study [26], demonstrating that the sample size used in each test exhibited power > 0.95 at alpha = 0.05.

## 3. Results

### 3.1. Assessment of Apatite Precipitation Using FTIR and SEM-EDX

**Figure 1:**
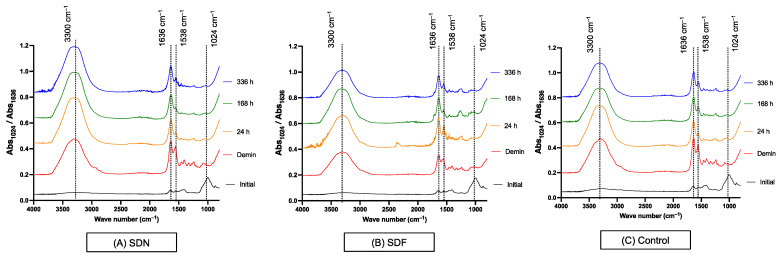
FTIR spectra of representative specimen from (**A**) SDN, (**B**) SDF, and (**C**) control groups at each time point.

**Figure 2:**
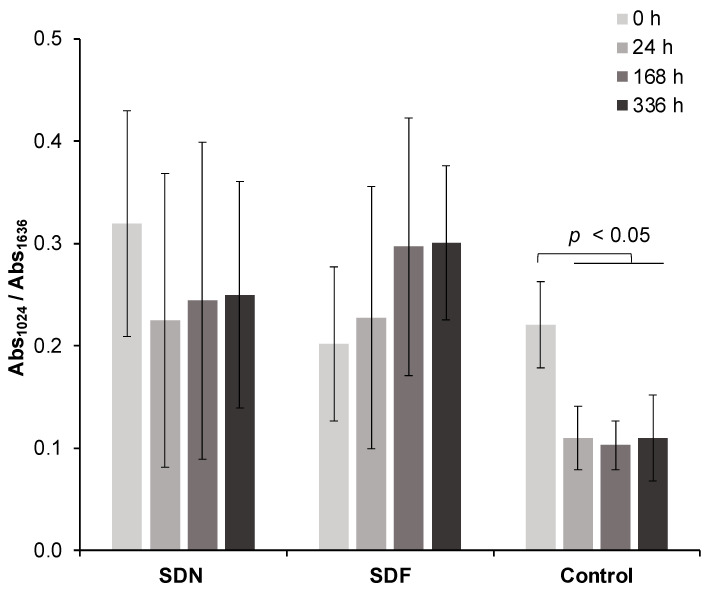
Abs_1024_/Abs_1636_ ratio of demineralized dentin specimens before and after immersing in simulated body fluid for 336 h (2 weeks). Error bars represent SD (*n* = 7), lines indicate *p-*values.

The FTIR spectra of the representative specimens at each time point are presented in Figure 1. A reduced phosphate peak (1024 cm^–1^) after demineralization was observed in all groups. The Abs_1024_/Abs_1636_ ratio of the control group at 0 h (0.22 ± 0.06) was significantly higher than that of specimens at 24 h (0.11 ± 0.04) (*p* = 0.0285), 168 h (0.10 ± 0.03) (*p* = 0.0148), and 336 h (0.11 ± 0.03) (*p* = 0.0377) (Figure 2). The mean Abs_1024_/Abs_1636_ ratio of the SDF group increased from 0.20 ± 0.10 at 0 h to 0.30 ± 0.16 at 336 h. The Abs_1024_/Abs_1636_ ratio of the SDN group at 0 h was 0.32 ± 0.15, which was gradually reduced to 0.25 ± 0.22 at 336 h. However, changes in the Abs_1024_/Abs_1636_ ratio for the SDF and SND groups at each time point were not significantly different (*p* > 0.05). Additionally, the ratio between SDF, SDN, and control groups at each time point was comparable (*p* > 0.05).

**Figure 3:**
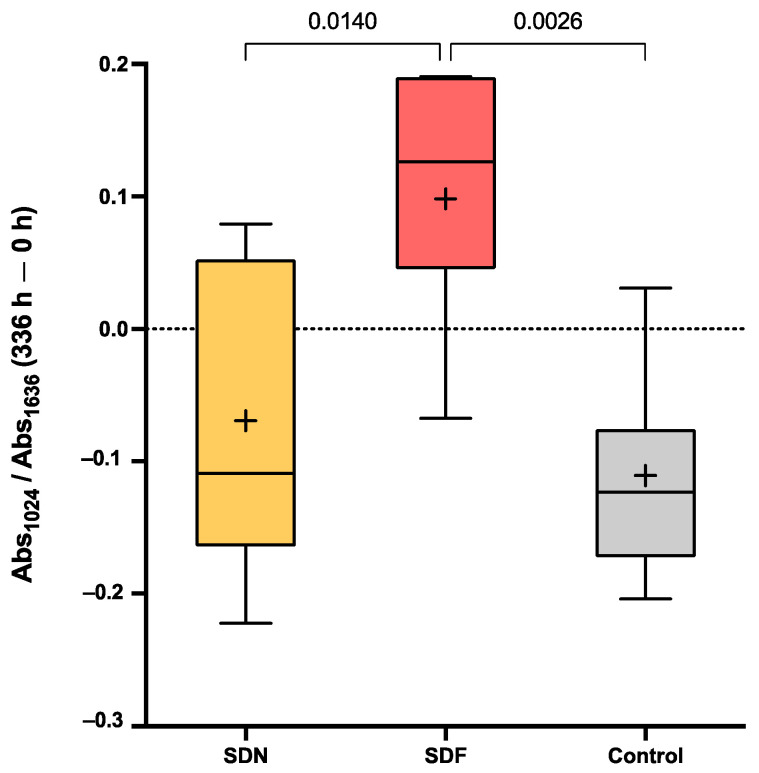
Box plots of differences in Abs_1024_/Abs_1636_ after 2 weeks compared with initial value (336 vs. 0 h). Boxes represent first quartile (Q1) to third quartile (Q3), horizontal lines in box represent median, and whiskers represent maximum and minimum values (*n* = 7). Lines indicate *p* < 0.05.

The difference in Abs_1024_/Abs_1636_ of the SDF group at 336 h compared with 0 h (0.10 ± 0.09) was significantly higher than that of the SDN (−0.07 ± 0.12) (*p* = 0.0140) and control (−0.11 ± 0.08) (*p* = 0.0026) groups (Figure 3). Additionally, the difference in the ratio was not significantly different between the SDN and control groups (*p* = 0.7175).

**Figure 4:**
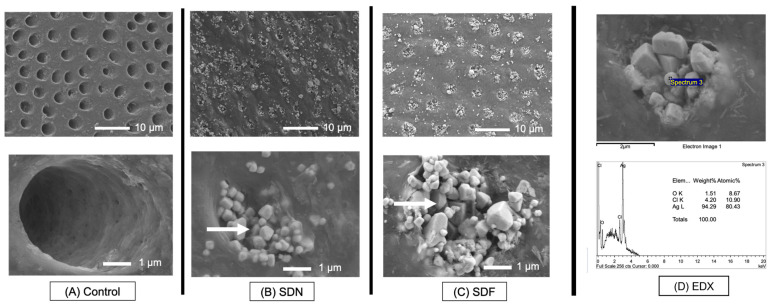
(**A**–**C**) SEM images at low and high magnification of representative specimen from each group after 2 weeks. Precipitation of crystals (arrows) occluding dentinal tubules was observed in SDN and SDF groups. (**D**) EDX result shows that crystals mainly contained Ag and Cl.

SEM images of the representative specimen of the control group show patent dentinal tubules (Figure 4A). SEM images of the SDN (Figure 4B) and SDF (Figure 4C) groups show crystals occluding dentinal tubules. The EDX results indicate that the precipitation observed in the SDN and SDF groups mainly contained Ag and Cl (Figure 4D).

### 3.2. Assessment of Mineral Precipitation Using Synchrotron-Based X-ray Tomography (SRXTM)

**Figure 5:**
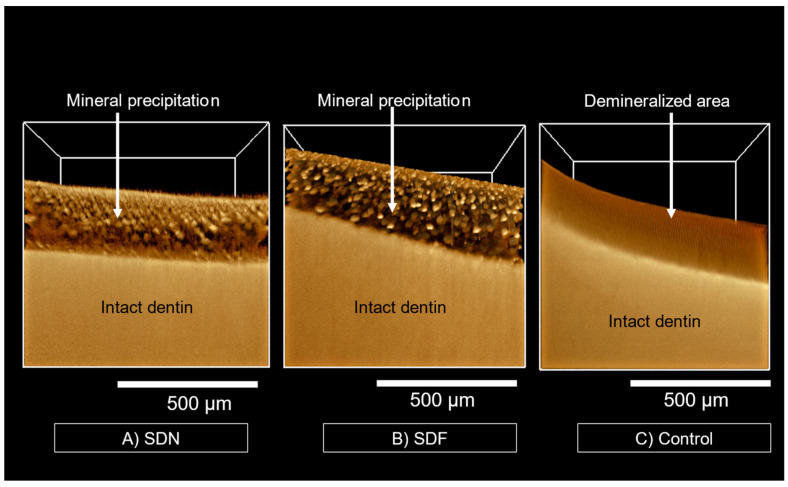
SRXTM images of representative specimen from each group after immersion in simulated body fluid for 2 weeks. (**A**,**B**) Mineral precipitation (arrows) was detected in SDN and SDF groups. (**C**) No precipitation was detected in control group. Three-dimensional images of specimens are provided in the Supplementary Materials (Videos S1–S3).

The SRXTM images of representative specimens of the SDN (Figure 5A, Video S1) and SDF (Figure 5B, Video S2) groups show multiple radiodense areas throughout the depth of the radiolucent area (~200 μm). No radiodense areas were detected in the specimen from the control group (Figure 5C, Video S3).

### 3.3. Cell Viability

The relative OD of the SDF group (92 ± 8%) was higher than that of the SDN group (88 ± 10%). However, the results were not significantly different (*p* = 0.5579).

## 4. Discussion

The aim of the current study was to compare the remineralizing and cytotoxic effects of silver diamine nitrate (SDN) and silver diamine fluoride (SDF). The increased Abs_1024_/Abs_1636_ ratio of the SDF group was significantly higher than that of the SDN and control groups. However, the mineral precipitation, as seen in SRXTM images, and the cytotoxicity of the SDN and SDF groups were comparable. Hence, the null hypothesis of the current study was partially rejected. It should be mentioned that study was an in vitro study; thus, the clinical relevance should be interpreted with caution.

The FTIR-ATR results of the current study indicate that the increase in peaks representing apatite formation was greater in the SDF group than the SDN group. This could be due to the effects of fluoride. It was demonstrated that fluoride can act as a catalyst for phosphate and calcium ion addition into the crystal lattice, thus promoting the growth of apatite crystals [43]. This may have subsequently promoted mineral apatite formation in the SDF specimen. Additionally, it was reported that the incorporation of fluoride into the lattice of remnant crystals promoted crystal transformation and reduced the solubility of apatite [44]. This study aimed to assess mineralization when the solution penetrates into the dentin and is exposed to dentinal fluid. However, it should be mentioned that a protocol for preparing simulated dentinal fluid has not yet been established. We speculated that the inorganic components of dentinal fluid would be similar to other types of body fluid [45]. Simulated body fluid (SBF) was therefore selected as the storage solution in the current study.

A high level of fluoride from SDF was expected to encourage the formation of low-soluble fluorohydroxyapatite (FHA) in the demineralized dentin. It is known that the precipitation of FHA after SDF application is not easily detected. This is in agreement with the current study, because the EDX failed to detect fluoride on the specimens. This could be due to the low sensitivity of EDX to detect small amounts of fluoride. Hence, the use of an alternative technique such as XRD, XPS, SAX/WAX, or Raman microcopy may be needed in future work to confirm the formation of fluorohydroxyapatite. Additionally, the application of SDF may encourage the precipitation of CaF_2_, which can act as a fluoride reservoir [46,47]. However, CaF_2_ globules were not detected in the SEM images of specimens. This could be due to the rapid washout of water-soluble CaF_2_ during rinsing with water [47,48].

The SEM images showed substantial mineral crystals occluding the patent dentinal tubules on the surface of specimens from the SDF and SDN groups. EDX showed that the crystals mainly contained Ag and Cl, indicating the formation of silver chloride salts. It is believed that Ag ions of SDF and SDN react with ions in the environment, producing silver phosphate (solubility of 6.4 × 10^−3^ g/100 mL) and silver oxide (1.3 × 10^−3^ g/100 mL), which are highly soluble. Then, the silver compounds may readily react with chloride in the environment, forming lower-soluble silver chloride (8.9 × 10^−5^ g/100 mL) [47,49]. The formation of silver chloride caused a black/metallic appearance on the tooth surface after SDF application. The formation of silver salts increased the hardness of dentin and blocked dentinal tubules, thus reducing the irritation on the pulp–dentin complex [9,47].

The use of synchrotron radiation X-ray tomographic microscopy (SRXTM) enabled 3D visualization of mineral precipitation at a higher resolution (pixel size ~1.44 μm) compared with conventional micro-CT (pixel size ~8 μm) [50]. The more radiodense areas in SRXTM images were expected to be silver salts [26]. The degree of mineral precipitation was similar in the SDN and SDF groups. This may be due to a comparable concentration of silver ions contained in both (25 ppm). However, the limitation of SRXTM is the risk of overestimating mineral density due to the high radiopacity of silver [51]. Additionally, the density of mineral crystals per volume may be affected by the dehydration and shrinkage of demineralized dentin layers. It should be mentioned that particles with a diameter smaller than the minimum resolution of the SRXTM (1.44 μm) were not detected in the images. This may lead to underestimation of the mineral density of small nanosized crystals such as hydroxyapatite in specimens [52].

The SDF solution can be rapidly adsorbed into dentin. Thus, the concern with applying SDF in deep cavities is the risk of toxic effects on dental pulp cells, leading to pulpal pain and inflammation. It has been shown that SDF reduces pulpal-like cell viability via the depletion of glutathione [13]. The use of high-molecular weight nitrate molecules in the solution was expected to lower the reactivity of the reactive ions, which could potentially reduce the toxic effects on pulp cells. However, the relative OD values of SDF and SDN groups were comparable. This could be due to the similar concentrations of silver ions in SDF and SDN in the current study. BS EN ISO 10993–5: 2009 (Biological evaluation of medical devices Part 5: Tests for in vitro cytotoxicity) states that a reduction in cell viability by more than 30% is considered a cytotoxic effect [53]. The current study demonstrated that cell viability was reduced after treatment with SDF and SDN by approximately 8% and 13%, respectively.

The limitation of the current study was that the cells were treated with a single concentration of SDF or SDN, which may not represent the clinical situation. The concentration of the solution may be reduced or diluted upon penetration through dentin. It was reported that the concentration of silver ions in dentin was reduced from 1.7 to 0.3 wt % at 20 μm depth [12]. The actual concentration of ions at the pulpal region may be much lower than that used in the current study. Hence, a more relevant model, such as a dentin penetration test, which contains a dentin barrier over the pulp cells, should be used in future studies [54]. Additionally, future work could examine the remineralizing effects of SDN or AgNO_3_ combined with NaF [17,18]. This could help provide alternative options for materials to control caries when SDF is not available.

## 5. Conclusions

In this study, we compared the in vitro remineralizing action between silver diamine nitrate (SDN) and silver diamine fluoride (SDF). The use of SDF provided a superior increase in apatite formation compared with SDN. However, the precipitation of silver salts occluding dentinal tubules in demineralized dentin observed with SDF and SDN was comparable. Additionally, the cytotoxic effects on dental pulp cells with SDN were not significantly different compared to SDF. SDN may be considered as an alternative material to control caries. However, more clinical studies are needed to confirm the anti-cavity action of the material.

## Figures and Tables

**Table 1:** Chemicals used to prepare SBF in the current study. All chemicals were purchased from Sigma Aldrich (St. Louis, MO, USA).

Order	Chemical	Amount (g)
1	NaCl	8.035
2	NaHCO_3_	0.355
3	KCl	0.225
4	K_2_HPO_4_·3H_2_O	0.231
5	MgCl_2_·6H_2_O	0.311
6	HCl (1 M)	38
7	CaCl_2_·2H_2_O	0.386
8	Na_2_SO_4_	0.072
9	Tris, NH_2_C(CH_2_OH)_3_	6.118

**Table 2:** Concentration (10^−3^ mol) of ions in SBF and blood plasma.

Ion	SBF (pH 7.4)	Blood Plasma (pH 7.2–7.4)
Na^+^	142.0	142.0
K^+^	5.0	5.0
Mg^2+^	1.5	1.5
Ca^2+^	2.5	2.5
Cl^−^	147.8	103.0
HCO_3_^−^	4.2	27.0
HPO_4_^2−^	1.0	1.0
SO_4_^2−^	0.5	0.5

## Data Availability

The datasets generated and/or analyzed during the current study are available from the corresponding author upon reasonable request.

## Supplementary Materials

The following supporting information can be downloaded at: www.mdpi.com/article/10.3390/jfb13010016/s1, Video S1: Representative specimen of control group shows no mineral precipitation. Videos S2 and S3: Representative specimens of SDN and SDF groups, respectively. Mineral precipitation is indicated in green.
